# Integrative analysis of the mouse fecal microbiome and metabolome reveal dynamic phenotypes in the development of colorectal cancer

**DOI:** 10.3389/fmicb.2022.1021325

**Published:** 2022-09-28

**Authors:** Jingjing Liu, Mingyang Qi, Chengchao Qiu, Feng Wang, Shaofei Xie, Jian Zhao, Jing Wu, Xiaofeng Song

**Affiliations:** ^1^Department of Biomedical Engineering, Nanjing University of Aeronautics and Astronautics, Nanjing, China; ^2^State Key Laboratory of Translational Medicine and Innovative Drug Development, Jiangsu Simcere Pharmaceutical Co., Ltd., Nanjing, China; ^3^School of Biomedical Engineering and Informatics, Nanjing Medical University, Nanjing, China

**Keywords:** gut microbiota, microbiome, metabolome, colorectal cancer, inflammation

## Abstract

The gut microbiome and its interaction with host have been implicated as the causes and regulators of colorectal cancer (CRC) pathogenesis. However, few studies comprehensively investigate the compositions of gut bacteria and their interactions with host at the early inflammatory and cancerous stages of CRC. In this study, mouse fecal samples collected at inflammation and CRC were subjected to microbiome and metabolome analyses. The datasets were analyzed individually and integratedly using various bioinformatics approaches. Great variations in gut microbiota abundance and composition were observed in inflammation and CRC. The abundances of *Bacteroides*, *S24-7_group_unidifineted*, and *Allobaculum* were significantly changed in inflammation and CRC. The abundances of *Bacteroides* and *Allobaculum* were significantly different between inflammation and CRC. Furthermore, strong excluding and appealing microbial interactions were found in the gut microbiota. CRC and inflammation presented specific fecal metabolome profiling. Fecal metabolomic analysis led to the identification and quantification of 1,138 metabolites with 32 metabolites significantly changed in CRC and inflammation. 1,17-Heptadecanediol and 24,25,26,27-Tetranor-23-oxo-hydroxyvitamin D3 were potential biomarkers for CRC. 3α,7β,12α-Trihydroxy-6-oxo-5α-cholan-24-oic Acid and NNAL-N-glucuronide were potential biomarkers for inflammation. The significantly changed bacterial species and metabolites contribute to inflammation and CRC diagnosis. Integrated microbiome and metabolomic analysis correlated microbes with host metabolites, and the variated microbe-metabolite association in inflammation and CRC suggest that microbes facilitate tumorigenesis of CRC through interfering host metabolism.

## Introduction

Colorectal cancer (CRC) ranks third in the mortality rate of malignant tumors, affecting more than a quarter of the world’s population (Brenner et al., 2014). It is now commonly believed that chronic inflammation is responsible for the occurrence of neoplastic transformation of the intestinal epithelium (Ullman and Itzkowitz, 2011). Inflammation promotes tumor outgrowth in the overlying epithelium and was the critical factor for CRC development (Clevers, 2004). Understanding the cell microenvironment in early inflammatory and cancerous stages of CRC is useful for its early diagnosis and treatment.

In recent years, a large number of emerging data indicated that gut microbiota has been deemed as a key environmental factor contributing to the progression of CRC through microbial metabolites, energy balance disturbance, and inflammatory response (Song et al., 2015; Plummer et al., 2016; Wong and Yu, 2019). Gut microbiota disorder altered the gut ecosystem and has been implicated in changes in CRC. Thus, the gut microbiota has come to the forefront as a reflection of the tumor environment. Nucleic acid sequencing of the bacterial 16S rRNA gene remains the most widely used stable target for bacterial identification and genetic evolutionary studies, and was widely used in human bacterial pathogens identification (Church et al., 2020). For example, based on 16S rRNA sequencing, it was found that *F. nucleatum* could generate a pro-inflammatory environment for colorectal neoplasia progression in ApcMin mice (Kostic et al., 2013).

Intestinal metabolites are important factors regulating and reflecting pathological processes of CRC as well. For example, some nitrogenous metabolites have the potential to promote cancer and exert carcinogenic effects *via* DNA alkylation, which can cause mutations (Gill and Rowland, 2002). Metabolomics could be applied to uncover the host gut metabolome to explain the diet or disease impacts on intestinal metabolism (Wu et al., 2016; Wang et al., 2020). Combined microbiome and metabolomics analysis will provide an alternative approach to study the CRC progression through associated alternations in the gut environment (Ji et al., 2019; Yachida et al., 2019; Chen et al., 2022; Yang et al., 2022). The microbiome and the metabolome in intestine could be the robust non-invasive targets for precision medicine.

Although there are a few studies showed associations between gut microbiota and CRC, the profile of gut microbial community and their impact on host metabolism at the initial inflammatory and cancerous stages of CRC remain unclear. Furthermore, the interplay between gut microbiota and intestinal metabolites in inflammation and CRC has not been comprehensively investigated. Thus, we built an inflammation-associated colorectal cancer model in mouse. The fecal microbiome and metabolome at inflammation and CRC were studied to obtain evidence of dynamic phenotypes of fecal microorganisms and metabolites.

## Materials and methods

### Animal experiments and sample collection

**FIGURE 1:**
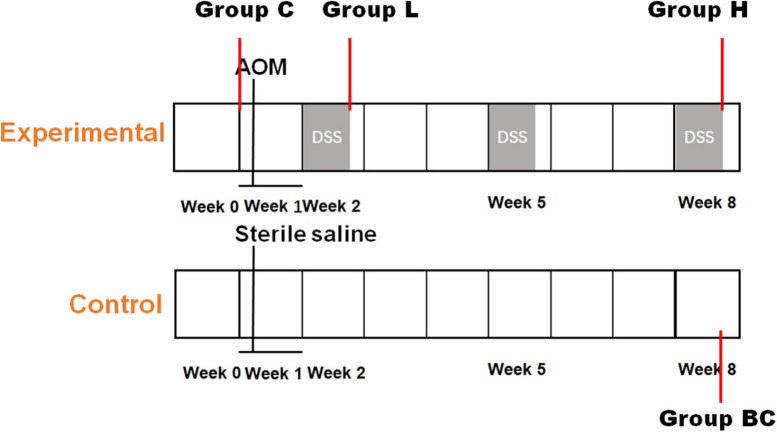
Establishment of mouse colorectal cancer model. Timeline of chemical dosage in control (bottom) and experimental (top) mice.

Eight 4-weeks-old C57BL/6 mice were obtained from Nanjing Medical University (SPF grade, SCXK 2016–0002) and approved by the experimental animal administration committee of Jiangsu Simcere pharmaceutical Co., Ltd. (approval No. 011). Mice were evenly divided into two groups, control and experimental groups, each group had four replicates. After 1 week of acclimatization, mice in the control group were intraperitoneally injected with normal saline. While mice in the experimental group was intraperitoneally injected with azoxymethane (AOM, 12.5 mg/kg). One week later, mice of experimental group would undergo intermittent oral administration of dextran sulfate sodium salt (DSS). DSS (2.5%, w/w) was added to the drinking water of mice at week 2, week 5, and week 8 (Figure 1). While in the other weeks, mice were fed with normal drinking water. The DSS-water feeding circle will be conducted for 9 weeks. Mice in the control group were fed with normal drinking water during the entire experiment. Fecal samples of experimental group were collected before AOM and DSS treatment (C group), after the first and last DSS administration cycle in week 2 (L group) and week 8 (H group). Feces collected from control group at the end of week 8 was regarded as BC group. Each Sunday, serum samples were also collected to evaluate the degree of inflammation using LC-MS/MS and MDA Assay Kit (Li et al., 2021). At the end of the experiment, the mice were all euthanized, and the colorectal tissues were collected and analyzed by hematoxylin and eosin (HE) staining.

### Liquid chromatography–tandem mass spectrometry

Serum samples were analyzed by LC-MS/MS and MDA assay kit to evaluate the level of inflammation. The details of LC-MS/MS method were described. The serum samples were derivatized with fluorenylmethyloxycarbonyl chloride, and the N6-FMoc-lysine in samples were quantified by LC-MS/MS technology. N6-FMoc-D-lysine was purchased from TCI (Shanghai, China), and was quantified using an Agilent 1200 HPLC-coupled with an API 4500 QTRAP tandem mass spectrometer (AB SCIEX, USA). The LC-MS/MS method was built for N6-FMoc-D-lysine quantification. The fragment ion combination 369.4/175.1 showed the best sensitivity and specificity under 10 declustering potential and 20 collision voltage. C18 column (ACQUITY UPLC BEH C18, 1.7 μm, 2.1 × 50 mm, Waters) was used. The LC gradient was showed as followings. The flow rate was set at 0.4 ml/minute, and the injection volume was 10.0 μl. Mobile phase A was ddH_2_O with 0.1% formic acid, mobile phase B was acetonitrile. The gradient began at 5% solvent B, increased to 20% in 0.5 min, 20–95% in 3 min, 95–5% in 0.5 min, retained at 5% B for 1 min.

### Fecal DNA and metabolites extraction

QIAamp DNA Stool Mini Kit (Qiagen, Hilden, Germany) was used to extract DNA from fecal samples. The extraction procedure was conducted according to manufacturer’s guidelines. DNA purity and integrity were verified by liquid chromatography (Agilent, USA) and 1% agarose gel electrophoresis. The concentration of DNA was determined by NanoDrop spectrophotometry (NanoDrop, Germany).

Generally, 50 mg fecal sample was lyophilized in 400 μl extraction buffer (methanol/ddH_2_O = 4:1), then a steel ball was added. The sample mixture was grinded for 6 min and sonicated at 5^°^C for 30 min. Then the mixture was kept at −20^°^C for 30 min and centrifuged at 13,000 g and 4^°^C for 15 min. The supernate was collected and freeze dried. The dried sample was redisolved in 100 μl 90% methanol aqueous solution. Quality control sample was prepared by tanking 20 μl solution from each sample and mixed together. QC samples were injected at the interval of four to monitor and overcome analytical drifts at regular intervals in UPLC–MS/MS during the experimental sequence.

### 16S ribosomal RNA gene sequencing

The V3 to V4 region of the 16S ribosomal RNA (rRNA) gene was amplified with primer 338F (5′-ACTCCTACGGGAGGCAGCAG-3′) and 806R (5′-GGACTACHVGGGTWTCTAAT-3′) (Bao et al., 2022). Polymerase chain reaction (PCR) cycles were performed as follows: initial denaturation at 95^°^C for 3 min, followed by 27 cycles of heat and cooling, 95^°^C for 30 s, 55^°^C for 30 s, 72^°^C for 45 s, and kept at 72^°^C for 10 min. The whole sequencing process was conducted by Shanghai Meiji Biomedical Technology Co., Ltd. (Shanghai, China) using an ABI GeneAmp^®^ 9700 platform.

### Sequencing data analysis

Cutadapter (v1.10) was used to process our raw sequence reads (Martin, 2011). FastQC (v0.11.9) was applied to evaluate data quality (Wingett and Andrews, 2018). UCHIME2 was used to remove the chimera in the sequences (Edgar, 2016), then UCLUST was used to cluster the sequences into operational taxonomic units (OTUs) with 97% similarity (Edgar, 2010), the taxonomic classification was assigned by RDP classifier (v2.2) (Wang et al., 2007) against the Greengene database (v.13_8) (McDonald et al., 2012). Alpha diversity and beta diversity were performed to identify the complexity and diversity in samples. Principle coordinate analysis (PCoA) was conducted using weighted UniFrac distance metrics. The dissimilarities between groups was illustrated by the analysis of similarities (ANOSIM).

### LC-MS/MS data acquisition and analysis

Liquid chromatography-tandem mass spectrometry (LC-MS/MS) was performed as described (Wu et al., 2020). Samples were analyzed with UPLC-Triple TOF mass spectrometer (AB SCIEX 5600). Both HILIC column (ACQUITY UPLC BEH HILIC, 1.7 μm, 2.1 × 50 mm, Waters) and C18 column (ACQUITY UPLC BEH C18, 1.7 μm, 2.1 × 100 mm, Waters) were used. The flow rate was set at 0.4 ml/minute, and the injection volume was 10.0 μl. When it is HILIC column, the mobile phase consisted of two components: (A) acetonitrile/water (95/5, v/v) with 10 mM ammonium acetate and 0.1% formic acid, (B) acetonitrile/water (50/50, v/v) with 10 mM ammonium acetate and 0.1% formic acid. The gradient began at 0% solvent B, increased to 25% in 1 min, 25–40% in 3 min, 40–90% in 1 min, retained at 90% B for 1 min, followed by 2 min 100% solvent A. For C18 column, mobile phase A was ddH_2_O with 0.1% formic acid, mobile phase B was acetonitrile/isopropanol (50/50, v/v) with 0.1% formic acid. The gradient began at 5% solvent B, increased to 20% in 3 min, 20–95% in 6 min, 95–5% in 4 min, retained at 5% B for 3 min. The parameters of MS in positive ionization mode were applied for both chromatographic modes: IonSpray Voltage to 5,000 V; Ion Source Gas flow to 50 L/h; Curtain Gas to 30 L/h, Source Temperature to 500^°^C; Declustering Potential to 80 V; Collision Energy rolling from 20 to 60 V. MS data was obtained by data-dependent acquisition (DDA) and the same setting for both HILIC and C18 analysis. The mass range for both TOF-MS scan and Product Ion scan was set at 50–1,000 mass/charge (m/z).

The raw data files were processed by Progenesis QI (Waters Corporation, Milford, USA) for peak picking and alignment. Metabolite peaks were assigned by MS/MS analysis combined with the MassFragment™ application manager (Waters Corporation, Milford, USA) by way of chemically intelligent peak-matching algorithms and interpreted with available biochemical databases, such as the KEGG,^1^ Human Metabolome Database (HMDB)^2^, and METLIN^3^ (Kanehisa, 1997; Guijas et al., 2018; Wishart et al., 2018). Biomarker analysis was conducted on Metaboanalyst 5.0 (Pang et al., 2021).

### Statistical analysis

Significantly changed microorganisms and metabolites were evaluated with Welch’s *t*-test. Differences were deemed as significant when *p* < 0.05. Analysis of similarities (ANOSIM) was used to evaluated the group variations which described by PCoA analysis. Pearson correlation analysis was conducted to calculate the correlation between microorganisms or between metabolites and microorganisms. The *p*-value was adjusted by the Benjamini-Hochberg (BH) correction for a maximum 0.05 probability of false positive detection. All data were analyzed with R version 4.0.0 (R Foundation for Statistical Computing, Vienna, Austria).

## Results

### Inflammation assessment

**FIGURE 2:**
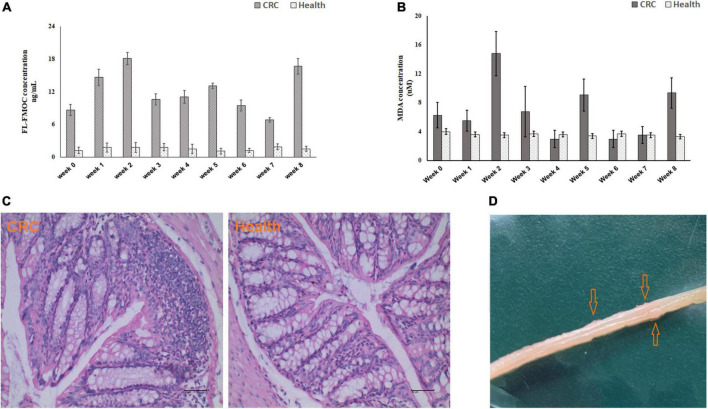
Biochemical testing of established mouse colorectal cancer model. Inflammation level detected by LC-MS/MS **(A)** and MDA Assay Kit **(B)** in control and experimental mice from week 0 to week 8. **(C)** HE staining of colorectal tissue in control (left) and experiment (right) group. **(D)** Macroscopic colorectal tumors in experimental mice.

Mice were injected with AOM and fed DSS to induce CRC (Figure 1). The level of inflammation was evaluated by detecting oxidative stress biomarker and lipid peroxidation in serum samples using LC-MS/MS and MDA Assay Kit, respectively. As shown in Figures 2A,B, according to the chemical administration cycle, the level of inflammation changed periodically. The level of inflammation increased in mice after the chemical dosage. Furthermore, HE staining of colorectal tissue indicated that sever inflammation occurred in the chemical dosed mice (Figure 2C). In the end, tumors were also observed on the intestinal surface of chemical dosed mice (Figure 2D). Therefore, long term inflammation in intestine will cause CRC.

### Diversity and composition of gut microbiota in inflammation and colorectal cancer

**FIGURE 3:**
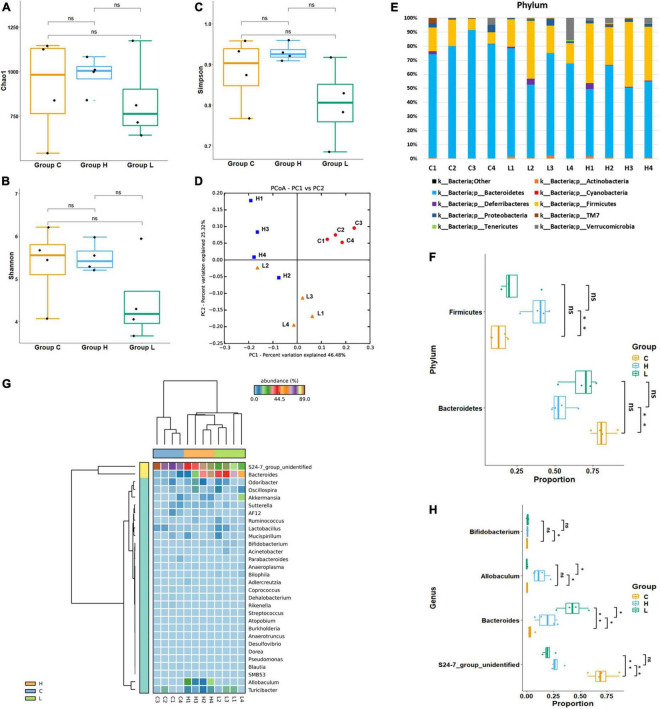
Diversity and abundancy analysis of gut microbiota. **(A)** Boxplots of Chao1 Richness Index. **(B)** Boxplots of Shannon Diversity Index. **(C)** Boxplots of Simpson Diversity Index. **(D)** PCoA plot of Group C, Group L, and Group H, showed a significant difference between health, colorectal inflammation, and colorectal cancer. **(E)** Relative abundance of microbial communities at phylum level. The relative abundance is defined as a percentage of the total microbial sequences in a sample. **(F)** Boxplots of significantly changed floras at phylum level. **(G)** Heat map of the 37 most abundant genera at genus level. **(H)** Boxplots of significantly changed floras at genus level. ns: *p* > 0.05, no significance. *p ≤ 0.05; **p ≤ 0.01.

In total, 318,842 16S rRNA reads from 16 fecal samples were obtained. The average number of reads per sample in group BC, C, L, and H were 18,206, 21,317, 20,998, and 19,189. We generated OTUs at 97% similarity level and the total number of OTUs in group C, L, and H was 7,221, the OTUs number of each sample was shown in Table 1. The α diversity and β diversity between group BC and C were compared, and no significant difference was observed, which demonstrated that the gut microbiota stayed stable during the normal growth of mice (Supplementary Figures 1, 2). Thus, the variations in the diversity and composition of gut microbiota between group C, L, and H were caused by the pathological process of CRC. The α diversity of the gut microbiota in health, inflammation and CRC were depicted using Chao1 Index (Figure 3A), Shannon Index (Figure 3B), and Simpson Index (Figure 3C). There were no significant differences in the Chao1 Index (group C, 912 ± 284; group L, 834 ± 235; group H, 982 ± 102), Shannon Index (group C, 5.3 ± 0.9; group L, 4.4 ± 1.0; group H, 5.5 ± 0.3), and Simpson Index (group C, 0.88 ± 0.08; group L, 0.80 ± 0.10; group H, 0.92 ± 0.02). Principal coordinate analysis (PCoA) based on the weighted UniFrac distance metrics was performed to investigate the β diversity of gut microbiota in health, inflammation, and CRC. The result of PCoA analysis showed that samples from the same group clustered together and separated from the other, indicating that microbial community composition variated between different groups (Figure 3D). Similarity analysis demonstrated that microbial community compositions had significantly changed in group L and H compared with group C, which means inflammation and tumorigenesis of CRC caused great variations in gut microbiota community composition. (ANOSIM, Group C vs. Group L, *r* = 0.79, *p*-value = 0.03; Group C vs. Group H: *r* = 0.98, *p*-value = 0.03). There was no significant variation between group L and H, suggesting similar gut microbiota community composition in inflammation and CRC (ANOSIM, Group L vs. Group H, *r* = 0.40, *p* = 0.06). These results suggested that the composition of gut microbiota could be greatly altered by the pathological process of CRC.

**TABLE 1:** Statistics of sequencing and OTUs of 16S rRNA in Group C, L, and H.

Sample ID	Number of reads	Observed OTUs
BC1	16,254	699
BC2	28,522	682
BC3	21,147	816
BC4	21,531	479
C1	18,026	596
C2	24,602	776
C3	23,776	741
C4	18,864	378
L1	20,871	505
L2	18,748	810
L3	23,866	437
L4	20,509	458
H1	21,569	544
H2	22,877	628
H3	16,734	706
H4	15,576	642

### Alternations of gut microbiota associated with inflammation and colorectal cancer

At phylum level, *Bacteroidetes* had highest abundance in each group, and *Firmicutes* was the second most abundant phylum. Although *Bacteroidetes* and *Firmicutes* were the dominant bacteria in health, inflammation and CRC, their abundances have changed during the pathological process of CRC (Figure 3E). The average abundance of *Bacteroidetes* in Group C, Group L, and Group H were 81.5, 67.4, and 54.4%. The abundance of *Bacteroidetes* has decreased during the CRC progression. The average abundance of *Firmicutes* in Group C, Group L, and Group H were 13.0, 23.6, and 38.5%. The abundance of *Firmicutes* has increased during the CRC progression. Welch’s *t*-test was used to identify whether there were significant changes in the dominant bacteria due to the development of CRC (Figure 3F). Significant variations in the abundance of *Bacteroidetes* (*p* = 0.002) and *Firmicutes* (*p* = 0.003) were observed between group C and H, suggesting that *Firmicutes* booming and *Bacteroidetes* depression contribute to the development of CRC.

At genus level, *S24-7_group_unidifineted* and *Bacteroides* were dominant genera (Figure 3G). Welch’s *t*-test was used to evaluate whether there were significant differences in the abundance of genera between different groups (Figure 3H). The average abundance of *S24-7_group_unidifineted* in Group C, Group L and Group H was 70.2, 19.8, and 27.7%. The abundances of *S24-7_group_unidifineted* significantly decreased in inflammation and CRC, and the abundance of *S24-7_group_unidifineted* was significantly different between CRC and inflammation (Group C vs. Group L: *p*-value = 5.16 × 10^–5^, Group C vs. Group H: *p*-value = 1.55 × 10^–4^, Group L vs. Group H: *p*-value = 0.03). *Bacteroides*, a subclass of *Bacteroidaceae*, its average abundance was 3.6% in Group C, then increased to 42.7% in Group L and 18.8% in group H. *Bacteroides* had significantly changed in inflammation and CRC. Besides, significant variation in *Bacteroides* abundance was also observed between inflammation and CRC (Group C vs. Group L: *p*-value = 3.21 × 10^–3^, Group C vs. Group H: *p*-value = 0.04, Group L vs. Group H: *p*-value = 0.02). The average abundance of *Allobaculum* was 0.12% in Group C and 0.11% in Group L, and dramatically increased to 12.6% in Group H (Group C vs. Group H: *p*-value = 0.031, Group L vs. Group H: *p*-value = 0.031). Result from Welch’s *t*-test showed that the growth of *Allobaculum* was initially stable in inflammation and then massively expanded in CRC. The growth of *Bifidobacterium* was inhibited in inflammation and later recovered to its initial abundance in CRC (Group C vs. Group L: *p*-value = 0.04). Overall, *Bacteroides, S24-7_group_unidifineted*, *Allobaculum*, and *Bifidobacterium* were significantly changed in CRC, they could be used in CRC auxiliary diagnosis. The abundances of *Allobaculum* and *Bacteroides* were greatly different between inflammation and CRC, they could be used to distinguish early inflammation from CRC. The enriched metabolic pathways of changed gut bacteria were listed in Table 2.

**TABLE 2:** Significant changed pathways in different groups.

Pathway	Type	Alteration trend	Fold-change	*P*-value
Amino acid metabolism	L/C	**↓**	0.93	0.013
Cancers*	L/C	**↑**	1.28	0.034
Carbohydrate metabolism	L/C	**↑**	1.09	0.017
Cell growth and death	L/C	**↓**	0.91	0.027
Cellular processes and signaling	L/C	**↑**	1.15	0.017
Endocrine system	L/C	**↑**	1.18	0.037
Energy metabolism	L/C	**↓**	0.91	0.005
Enzyme families	L/C	**↓**	0.94	0.007
Metabolism	L/C	**↑**	1.07	0.027
Metabolism of cofactors and vitamins	L/C	**↓**	0.89	0.001
Nervous system	L/C	**↑**	1.14	0.007
Nucleotide metabolism	L/C	**↓**	0.89	0.011
Poorly characterized	L/C	**↑**	1.06	0.005
Replication and repair	L/C	**↓**	0.90	0.026
Transcription*	L/C	**↑**	1.27	0.006
Translation	L/C	**↓**	0.85	0.006
Folding, sorting, and degradation	H/C	**↓**	0.91	0.013
Amino acid metabolism	H/C	**↓**	0.93	0.034
Cell growth and death	H/C	**↓**	0.90	0.017
Energy metabolism	H/C	**↓**	0.90	0.027
Environmental adaptation	H/C	**↑**	1.12	0.017
Excretory system*	H/C	**↓**	0.77	0.037
Glycan biosynthesis and metabolism	H/C	**↓**	0.86	0.005
Membrane transport*	H/C	**↑**	1.33	0.007
Metabolic diseases	H/C	**↓**	0.85	0.027
Metabolism	H/C	**↑**	1.03	0.001
Metabolism of cofactors and vitamins	H/C	**↓**	0.88	0.007
Nervous system	H/C	**↑**	1.11	0.011
Nucleotide metabolism	H/C	**↓**	0.92	0.005
Poorly characterized	H/C	**↑**	1.05	0.026
Transcription	H/C	**↑**	1.25	0.006
Translation	H/C	**↓**	0.91	0.006
Carbohydrate metabolism	H/L	**↓**	0.95	0.043
Endocrine system	H/L	**↓**	0.86	0.047
Enzyme families	H/L	**↑**	1.05	0.042

*Significantly changed pathway related to colorectal cancer.

### Bacteria and bacterial interactions associated with inflammation and colorectal cancer

**FIGURE 4:**
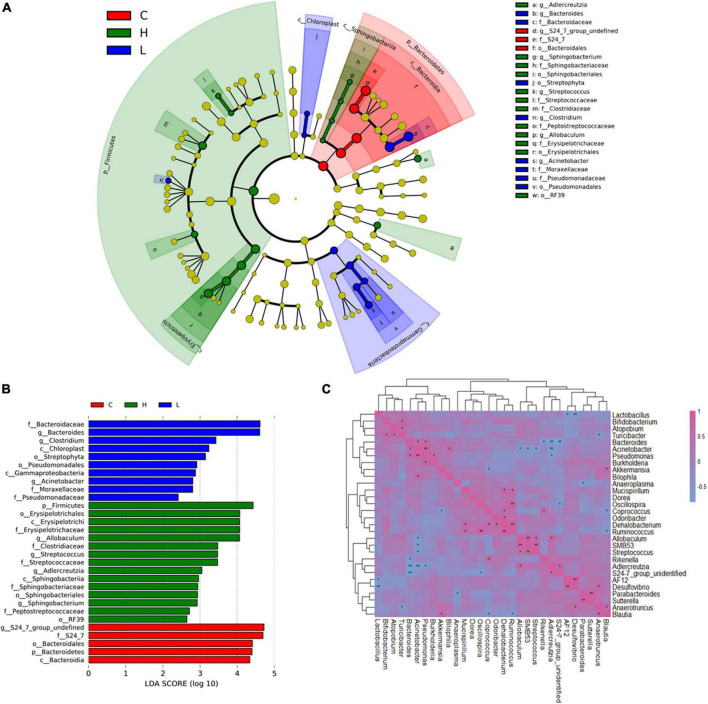
Linear discriminant analysis (LDA) effect size (LEfSe) and correlation analysis of bacterial communities based on 16S rRNA gene sequences. **(A)** Taxonomic distribution of bacterial groups significant for inflammation and CRC. **(B)** Histogram of the LDA scores computed for differentially abundant bacterial taxa between health, inflammation and CRC. **(C)** Correlation analysis of the 30 most abundant genera in Group C, Group L, and Group H. *0.01 < *p* ≤ 0.05; **p ≤ 0.01.

LEfSe (Segata et al., 2011) was used to generate a cladogram to identify the specific bacteria that was associated with inflammation or CRC. 30 discriminatory OTUs were identified as key discriminant features (Figure 4A). Significant overgrowth of *Allobaculunm* (LDA scores (log10) > 4) was observed in the feces of CRC mice. *Bacteroides* was the most abundant genus in the feces of inflamed mice (LDA scores (log10) > 4). *S24-7_group_ unidentified* had the largest proportions (LDA scores (log10) > 4) in the feces of health mice (Figure 4B).

In order to investigate the interactions of gut microbiota, spearman correlation analysis was conducted at genus level to draw the correlation matrix of 30 most abundant taxa in gut microbiota (*p* < 0.05) (Figure 4C). We focus on the correlation of bacteria that have greatly changed due to the inflammation and CRC (*r* > 0.7 and *p* < 0.05). At genus level, *Bacteroides* had a particularly strong excluding interaction with *S24-7_group_ unidentified* (*r* = −0.83, *p* = 1.7 × 10^–3^), *Adlercreutzia* (*r* = −0.79, *p* = 3.6 × 10^–3^) and *Rikenella* (*r* = −0.65, *p* = 0.02). *Bacteroides* and *Pseudomonas* were strongly and positively correlated (*r* = 0.65, *p* = 0.02). *Allobaculum* had strong positive correlations with *Adlercreutzia* (*r* = 0.65, *p* = 0.02) and SMB53 (*r* = 0.82, *p* = 1.2 × 10^–3^), and had negative correlations with *Acinetobacter* (*r* = −0.65, *p* = 0.016). *Bifidobacterium* had a positive correlation with *Turicibacter* (*r* = 0.62, *p* = 0.03). *S24-7_group_unidentified* was positively correlated with *Adlercreutzia* (*r* = 0.67, *p* = 0.02), it also had an excluding interaction with *Bacteroides* (*r* = −0.83, *p* = 1.7 × 10^–3^) and *Oscillospira* (*r* = −0.66, *p* = 0.02).

### Identification of discriminatory metabolites in inflammation and colorectal cancer

**FIGURE 5:**
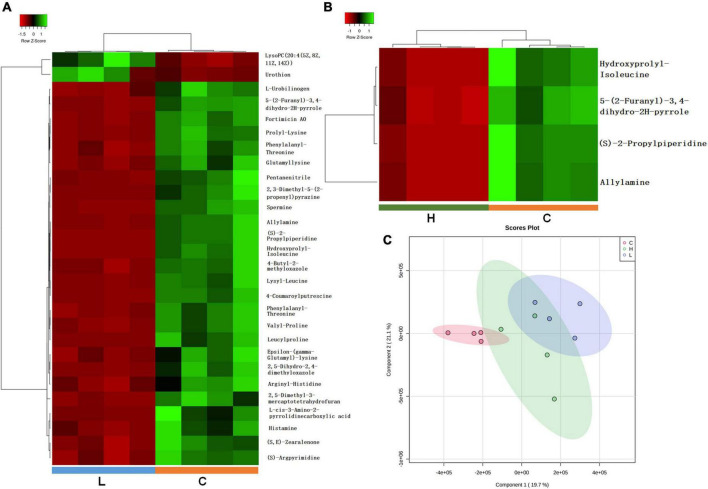
Important discriminatory metabolites identified by clustering, correlation, and multivariate analysis in inflammation and CRC. **(A)** Hierarchical clustering analysis (HCA) for the significantly changed metabolites in inflammation. **(B)** Hierarchical clustering analysis (HCA) for significantly changed metabolites in CRC. **(C)** OPLS-DA analysis displaying the grouped discrimination of health, inflammation and CRC by the first two PCs.

Gut microbiota was demonstrated to be associated with the development of CRC based on the microbiome analysis, we hypothesized that fecal metabolome may be partially affected due to gut microbiota alternations and CRC progression. Thus, metabolome analysis of fecal samples was conducted using LC-MS/MS based metabolomics approach. One thousand one hundred and thirty-eight metabolites were successfully identified and quantified in C, L, and H groups. Hierarchical clustering analysis on the metabolite abundances in the C, L and H group was performed (Supplementary Figure 3). Twenty-eight and thirty-eight metabolites significantly changed in the L group and H group, respectively (Supplementary Table 1). Pearson correlation analysis of metabolites showed correlations between significantly changed metabolites and phenotypes (Figures 5A,B).

**FIGURE 6:**
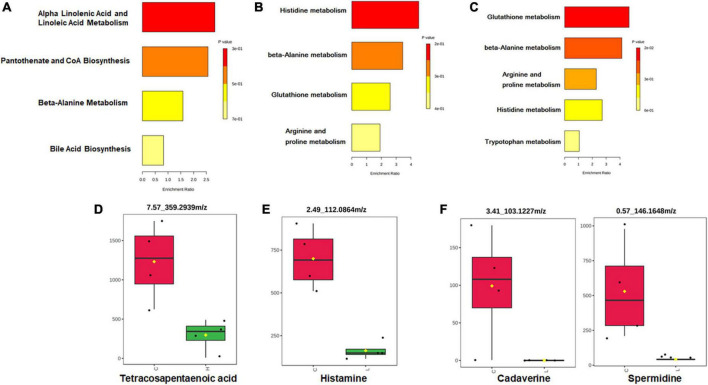
Pathway enrichment and statistical significance of the unique or significantly changed metabolites in CRC **(A)**, inflammation **(B)**, and health **(C)**. Represented metabolites engaged in the significantly changed metabolic pathways of CRC **(D)** and inflammation **(E)**. **(F)** Represented unique metabolites detected in health mice.

Partial Least Squares Discriminant Analysis (PLS-DA) was used to overview whether there were metabolic changes in CRC and inflammation (Figure 5C). Metabolome profiles of fecal samples within the same group clustered together and separated from the other, suggesting the obvious metabolic shifts in CRC and inflammation. Welch’s *t*-test was applied to find significantly changed metabolites in fecal samples of CRC and inflammation. Features with fold change > 2 and adjusted *p*-value < 0.05 (Benjamini-Hochberg) were considered significantly changed (Supplementary Figure 4). Significantly changed features were searched against databases (HMDB, LMSD, and KEGG) for metabolites identification. The identified metabolites and their library ID were listed in Supplementary Table 1, in total 66 significantly changed metabolites were characterized. There were 26 metabolites uniquely detected in health mice, 3 metabolites uniquely detected in inflamed mice and CRC mice (Supplementary Table 2). Moreover, we combined the significantly changed metabolites and unique metabolites for biological function analysis, and find out that the dysregulated metabolites in CRC were mainly engaged in alpha linolenic acid and linoleic acid metabolism (Figure 6A). The dysregulated metabolites in inflammation were engaged in histidine metabolism (*p* = 0.06) (Figure 6B). Unique metabolites detected in health mice were engaged in glutathione metabolism (Figure 6C). Represented metabolites engaged in the most significant metabolic pathways were showed. Tetracosapentaenoic acid was greatly down regulated in CRC mice (Figure 6D), concentration of histamine was significantly decreased due to inflammation (Figure 6E). Cadaverine and Spermidine were unique metabolites engaged in glutathione metabolism in health mice (Figure 6F). Taken together, our data clearly and robustly showed that CRC and inflammation presented specific fecal metabolome profiling.

### Correlations between microbes and metabolites in inflammation and colorectal cancer

**FIGURE 7:**
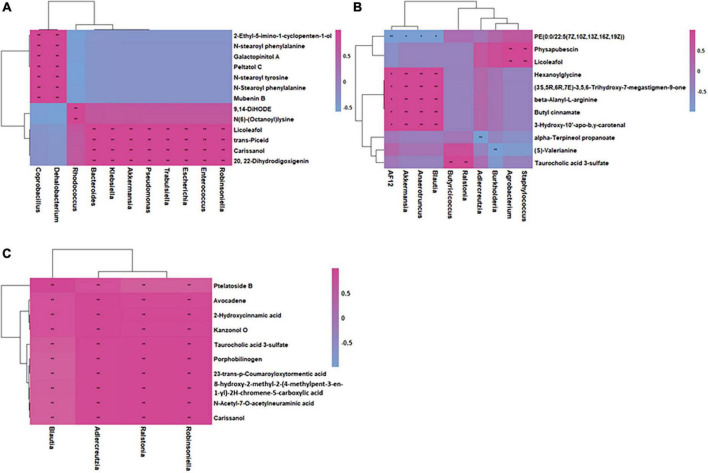
Integrated correlation-based network analysis (Pearson’s correlation) of microbes and metabolites in health **(A)**, inflammation **(B)**, and CRC **(C)**.

Based on the fecal microbiome and metabolomics data, we performed Pearson’s correlation analysis to identify microbe-associated metabolites in inflammation, CRC and health mice (Figures 7A–C). Compared with health mice, there were fewer significant strong metabolite-microbe correlations in inflamed mice and CRC mice, suggesting that interactions between metabolite and microbe were affected due to CRC progression. Three of the four microbes that significantly correlated to metabolites in CRC are also observed to be significantly associated with metabolites in inflammation. The microbe-metabolite correlations are much more similar between inflammation and CRC. It was found that same bacteria were associated with different metabolites in health and inflammation. While, common microbe-metabolite correlation was observed in inflammation and CRC. For instance, *Akkermansia* was correlated to different metabolites in inflammation and health. *Ralstonia* was correlated to the same metabolites in CRC and inflammation. In both inflammation and CRC, *Ralstonia* was strongly correlated to Taurocholic acid 3-sulfate (LMST05020031), a sulfated bile acid. According to Kim’s research, *Ralstonia* was able to cause renal injury (Kim et al., 2021). Sulfated bile acids were the metabolic products of cholestasis, the observed correlation between *Ralstonia* and Taurocholic acid 3-sulfate in our study suggests that *Ralstonia* induce renal problem probably through interfering bile acid metabolism. Furthermore, the association between *Ralstonia* and Taurocholic acid 3-sulfate predicts possible renal dysfunction during CRC development.

### Identification of colorectal cancer stage-specific metabolites

**FIGURE 8:**
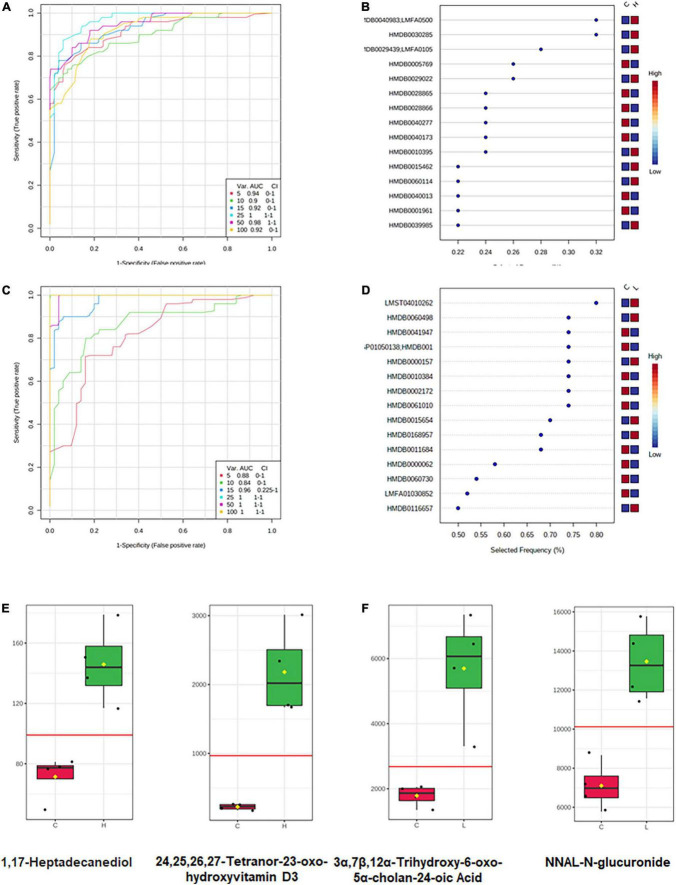
Metabolic biomarker analysis of CRC and inflammation. **(A)** ROC curve analyses based on random forests algorithms for biomarker analysis in CRC. **(B)** Top 15 metabolites discriminating CRC from health. **(C)** ROC curve analyses based on random forests algorithms for biomarker analysis in inflammation. **(D)** Top 15 metabolites discriminating inflammation from health. **(E)** Candidate metabolic biomarkers of CRC screened out by statistical analysis. **(F)** Candidate metabolic biomarkers of inflammation screened out by statistical analysis.

Different metabolome profiling was observed in CRC and inflammation, which could stand a chance for stage-specific metabolic biomarkers discovery. In order to predict the metabolic biomarkers, ROC (receiver operating characteristic) curve analyses were performed based on random forests algorithms. Top 15 most important metabolites for model construction was selected for candidate biomarker screening (Figures 8A–D). Among the 15 metabolites, metabolites with extremely higher concentrations in inflammation or CRC were considered as stage-specific candidate metabolites (Figures 8E,F). As a result, 1,17-Heptadecanediol and 24,25,26,27-Tetranor-23-oxo-hydroxyvitamin D3 were potential biomarkers for CRC. 3α,7β,12α-Trihydroxy-6-oxo-5α-cholan-24-oic Acid and NNAL-N-glucuronide were potential biomarkers for inflammation.

## Discussion

More and more evidences suggest that the gut microbiota contribute to tumorigenesis in CRC (Yachida et al., 2019; Wang et al., 2020). Thus, early screening and detecting carcinoma based on the gut microbiome is a promising field. A number of studies depicted the gut microbiota abundance and composition in chronic disease, and low gut bacterial richness was observed (Pascal et al., 2017). Consistently, in our study the gut bacteria richness obviously decreased when inflammation occurred and then gradually increased during the CRC development. Adaptation of gut microbiota to a long-term inflammatory environment results in a slight recovery in bacterial richness. Beta diversity analysis demonstrated that the composition of gut microbiota has greatly changed in CRC and inflammation. Significantly changed bacteria were observed in CRC and inflammation in the phylum and genus level. In general, the booming of *Firmicutes* and the decay of *Bacteroidetes* contribute to the tumorigenesis of CRC. Furthermore, *Bacteroides* was sensitive to both inflammation and CRC, its abundance was significantly increased in both inflammation and CRC. *Bacteroides* was previously observed with a high level in carcinoma and adenoma patients (Feng et al., 2015), and in our study, its abundance was proved also significantly increased in the initial inflammatory stage of CRC. Thus, *Bacteroides* could be a potential indicator for CRC early diagnosis. *Bacteroides, S24-7_group_unidifineted*, *Allobaculum* and *Bifidobacterium* were significantly changed in CRC, they could be used in CRC auxiliary diagnosis. *Allobaculum* and *Bacteroides* responded differently to inflammation and CRC, they could be used to distinguish inflammation from CRC.

The microbial interactions of significantly changed gut bacteria were investigated. *Bacteroides* had a particularly strong excluding interaction with *S24-7_group_ unidentified*. *Allobaculum* was strongly positively correlated with *SMB53* and *Streptococcus.* Based on the gut bacteria concentration variations during CRC development and the bacterial interactions, we could find out that the tumorigenesis of CRC would make *Bacteroides* and *Allobaculum* boom and occupy the intestinal tract, thereby inhibiting the growth of *S24-7_group_ unidentified*. Understanding the bacterial interactions during CRC development will provide more options for CRC treatment.

The fecal metabolome directly reflects interactions among dietary, environmental, and genetic factors. Thus, biomarkers may be more effectively identified through metabolomics of fecal samples. Following an untargeted approach, a larger number of metabolites that have greatly changed due to CRC development were identified in our study. Among the changed metabolites, most of them have a decreased concentration in inflammation and CRC. Previous studies have also documented a larger number of decreasing metabolites than increasing ones in the CRC patients (Monleon et al., 2009). In our study, histamine showed a lower concentration in inflammation than in the health mice, suggesting its involvement in tumorigenesis, which was demonstrated by other researchers that histamine regulates cancer-associated biological processes during cancer development in multiple cell types, including neoplastic cells and cells in the tumor micro-environment. Interestingly, tetracosapentaenoic acid had a lower concentration in the CRC than in the health mice. Further, we detected greater amounts of fatty acids, amino acids, peptides, and analogs in the health mice, which are known to be present in fecal samples from health adults (Cockbain et al., 2012; Dermadi et al., 2017). According to the pathway enrichment analysis results, the significantly changed metabolites and unique metabolites were engaged in variant pathways. Although tetracosapentaenoic acid and histamine showed good discrimination between disease mice and health mice, however, their concentrations are very low in both sick and healthy mice, which could bring burdens to their detection. Thus, biomarker analysis (Xia et al., 2015), an objective approach was conducted to find candidate biomarkers for CRC and inflammation. Concentrations of 1,17-Heptadecanediol and 24,25,26,27-Tetranor-23-oxo-hydroxyvitamin D3 were significantly increased in CRC and much higher than the detection limit, which make them good candidate biomarkers for CRC. Similarly, 3α,7β,12α-Trihydroxy-6-oxo-5α-cholan-24-oic acid and NNAL-N-glucuronide could be candidate biomarkers for inflammation.

The correlations between microbes and metabolites were investigated. The correlations of microbes and metabolites were different in inflammation, CRC, and health mice. While the microbe-metabolite correlations are much more similar in inflammation and CRC. This phenomenon suggested that interactions between gut microbiota and metabolites variated at different stages of CRC. Microbes probably facilitate CRC tumorigenesis through interfering host metabolism.

## Conclusion

Fecal microbiome data displayed the signature microbiota representing the CRC, inflammation and health status, i.e., enrichment of *Allobaculum*, *Bacteroids*, *Bifidobacterium* in CRC, *Allobaculum* and *Bacteroids* in inflammation. Furthermore, a non-targeted LC-MS-based metabolomics approach was applied to differentiate between health, inflammation and CRC, and associated different metabolites with specific phenotypes. Given that the study was conducted on mice, microbial and metabolic biomarkers in this study pending further validation studies. The integrated analysis of the identified microbes and fecal metabolites provides more functional insights than any single datasets. The variated microbe-metabolite associations in inflammation and CRC suggesting that microbes interfered host metabolism and facilitate tumorigenesis of CRC.

## Data availability statement

The microbiome sequcing data have been deposited on NCBI under BioProject ID PRJNA718119.

## Ethics statement

This animal study was reviewed and approved by the Animal Administration Committee of Jiangsu Simcere Pharmaceutical Co., Ltd.

## Author contributions

JL and JZ designed the study. JL, FW, and SX conducted the experiment. MQ and CQ analyzed the data. JL wrote the manuscript. JW and XS edited the manuscript. All authors contributed to the article and approved the submitted version.

## Footnotes

1
http://www.genome.jp/kegg/

2
http://www.hmdb.ca/

3
https://metlin.scripps.edu/
