# Supplementary material for: Integrative analysis of the mouse fecal microbiome and metabolome reveal dynamic phenotypes in the development of colorectal cancer
Source: Front Microbiol. 2022 Sep 28;13:1021325. doi: 10.3389/fmicb.2022.1021325 (PMC9554438; doi:10.3389/fmicb.2022.1021325)
Supplement: Supplementary file 1 [file Data_Sheet_1.PDF]

Table S1. Significantly changed metabolites in inflammation and CRC mice.

Feature ID	metabolite	Library ID	Log2 (FC)	p.adjusted	Type#
2.65_326.0370m/z	Urothion	HMDB0002377	2.71	0.048	L/C
4.19_145.1691m/z	(S)-2-Propylpiperidine	HMDB0030285	-4.38	0.039	L/C
3.92_156.1488m/z	Allylamine	HMDB0168957	-4.09	0.039	L/C
4.07_209.1278m/z	Hydroxyprolyl-Isoleucine	HMDB0028866	-3.82	0.039	L/C
4.65_312.1697m/z	5-(2-Furanyl)-3,4-dihydro-2H-pyrrole	HMDB0040013	-3.47	0.039	L/C
4.02_276.1909m/z	Prolyl-Lysine	HMDB0029022	-4.28	0.002	L/C
4.88_353.1788m/z	Fortimicin AO		-3.46	0.002	L/C
4.38_252.1702m/z	4-Coumaroylputrescine	HMDB0033461	-2.55	0.004	L/C
4.00_225.2062m/z	Spermine	HMDB0001256	-7.27	0.005	L/C
4.19_145.1691m/z	(S)-2-Propylpiperidine	HMDB0030285	-11.07	0.005	L/C
3.92_156.1488m/z	Allylamine	HMDB0168957	-7.25	0.005	L/C
3.46_256.1653m/z	Valyl-Proline	HMDB0029135	-3.73	0.005	L/C
4.98_284.1597m/z	Phenylalanyl-Threonine	HMDB0029005	-3.46	0.005	L/C
4.01_190.1427m/z	9-Oxo-nonanoic acid	HMDB0094711	-3.16	0.005	L/C
4.36_282.1807m/z	Lysyl-Leucine	HMDB0028955	-2.99	0.005	L/C
4.37_181.1325m/z	4-Butyl-2-methyloxazole	HMDB0037855	-2.94	0.005	L/C
3.77_237.1336m/z	(S)-Argpyrimidine	HMDB0037180	-2.59	0.006	L/C
4.07_209.1278m/z	Hydroxyprolyl-Isoleucine	HMDB0028866	-2.60	0.007	L/C
3.39_270.1804m/z	Leucylproline	HMDB0011175	-7.91	0.007	L/C
4.82_306.1566m/z	2,5-Dimethyl-3-mercaptotetrahydrofuran	HMDB0032237	-5.53	0.009	L/C
4.69_321.1684m/z	L-Urobilinogen	HMDB0004157	-3.76	0.009	L/C
4.05_293.1841m/z	Glutamyllysine	HMDB0004207	-3.09	0.010	L/C
4.95_336.1774m/z	(S,E)-Zearalenone	HMDB0031752	-2.04	0.011	L/C
4.49_214.1538m/z	Propenyl-L-NIO		-2.65	0.013	L/C
4.34_166.1331m/z	2,3-Dimethyl-5-(2-propenyl)pyrazine	HMDB0039985	-6.18	0.014	L/C
3.70_208.1796m/z	Pentanenitrile	HMDB0040173	-4.60	0.014	L/C
4.19_262.1524m/z	2,5-Dihydro-2,4-dimethyloxazole	HMDB0040518	-4.40	0.017	L/C
2.49_112.0864m/z	Histamine	HMDB0000870	-2.10	0.024	L/C
1.87_544.3370m/z	LysoPC(20:4(5Z,8Z,11Z,14Z))	HMDB0010395	1.80	0.027	L/C
2.24_276.1538m/z	Arginyl-Histidine	HMDB0028711	-3.07	0.031	L/C
5.03_258.1437m/z	Epsilon-(gamma-Glutamyl)-lysine	HMDB0003869	-3.14	0.032	L/C
2.49_95.0599m/z	L-cis-3-Amino-2-pyrrolidinecarboxylic acid	HMDB0029396	-3.35	0.043	L/C
4.19_145.1691m/z	(S)-2-Propylpiperidine	HMDB0030285	-4.38	0.039	H/C

3.92_156.1488m/z	Allylamine	HMDB0168957	-4.09	0.039	H/C
4.07_209.1278m/z	Hydroxyprolyl-Isoleucine	HMDB0028866	-3.82	0.039	H/C
4.65_312.1697m/z	5-(2-Furanyl)-3,4-dihydro-2H-pyrrole	HMDB0040013	-2.58	0.039	H/C
0.34_295.2153m/z	N-Acetylprocainamide	HMDB0041944	1.73	NA	H/C
0.34_432.2178m/z	Acidissiminol epoxide	HMDB0040793	1.51	NA	H/C
0.41_389.2656m/z	1alpha-hydroxy-25,26,27-trinorvitamin D3 24-carboxylic acid	LMST03020022	1.83	NA	H/C
0.89_402.1938m/z	Kanzonol N	HMDB0041100	0.59	NA	H/C
0.94_423.2735m/z	3α,7β,12α-Trihydroxy-6-oxo-5α-cholan-24-oic Acid	LMST04010262	2.65	NA	H/C
1.15_274.2732m/z	C16 Sphinganine	LMSP01040001	1.51	NA	H/C
1.33_376.2838m/z	24,25,26,27-Tetranor-23-oxo-hydroxyvitamin D3	HMDB0060114	9.75	NA	H/C
1.95_326.1948m/z	2-Dodecylbenzenesulfonic acid	HMDB0031031	0.64	NA	H/C
11.94_610.5756m/z	Cer(d16:1(4E)/22:0(2OH))	LMSP02010085	3.02	NA	H/C
3.06_329.1578m/z	1,7-Dimethylguanosine	HMDB0001961	0.45	NA	H/C
3.25_514.2690m/z	N-Docosahexaenoyl phenylalanine	HMDB0062333	0.45	NA	H/C
4.56_280.2011m/z	Val-Val-Val	HMDB0094676	0.41	NA	H/C
5.72_426.3203m/z	3alpha,7alpha,12beta-Trihydroxy-5beta-cholanoic acid	HMDB0000312	0.59	NA	H/C
6.61_375.2881m/z	1alpha,24-dihydroxy-25,26,27-trinorvitamin D3	LMST03020023	0.39	NA	H/C
6.97_310.2140m/z	13S-HpOTrE(gamma)	LMFA02000112	2.04	NA	H/C
7.00_368.2553m/z	Carboprost Tromethamine	HMDB0014573	1.53	NA	H/C
7.18_368.2560m/z	Misoprostol	HMDB0015064	1.79	NA	H/C
7.46_468.3674m/z	Coprocholic acid	HMDB0000601	0.39	NA	H/C
7.57_359.2939m/z	Tetracosapentaenoic acid	HMDB0006323	0.24	NA	H/C
7.95_525.2852m/z	LysoPE(0:0/22:6(4Z,7Z,10Z,13Z,16Z,19Z))	HMDB0011496	1.56	NA	H/C
7.96_543.3319m/z	PC(0:0/20:4(5Z,8Z,11Z,14Z))	LMGP01050121	1.81	NA	H/C
8.11_367.3677m/z	Pipercitine	HMDB0039937	0.65	NA	H/C
8.52_453.2852m/z	PE(16:0/0:0)	LMGP02050002	0.54	NA	H/C
8.56_328.2615m/z	MG(16:1(9Z)/0:0/0:0)	HMDB0011565	2.03	NA	H/C

11.47_537.5113m/z	Cer(d18:1/16:0)	HMDB0000790	1.71	NA	H/C
1.83_219.1102m/z	Pantothenic acid	HMDB0000210	0.27	NA	H/C
7.31_435.3456m/z	3a,7a,12a-Trihydroxy-5b-cholestan-26-al	HMDB0003533	0.53	NA	H/C
1.87_544.3370m/z	LysoPC(20:4(5Z,8Z,11Z,14Z)/0:0)	HMDB0010395	3.04	NA	H/C
1.97_522.3543m/z	LysoPC(18:1(11Z)/0:0)	HMDB0010385	1.66	NA	H/C
7.96_351.2524m/z	Cortol	HMDB0003180	2.22	NA	H/C
5.01_392.2023m/z	Paramethasone	HMDB0015462	0.14	NA	H/C
2.28_388.1592m/z	Aucubin	HMDB0036562	1.75	NA	H/C
2.67_513.3055m/z	Kukoamine D	HMDB0060527	0.14	NA	H/C
4.88_353.1788m/z	Fortimicin AO	C17972(KEGG)	0.22	NA	H/C

Type means the comparison between which two groups.
