# Supplementary material for: Integrative analysis of the mouse fecal microbiome and metabolome reveal dynamic phenotypes in the development of colorectal cancer
Source: Front Microbiol. 2022 Sep 28;13:1021325. doi: 10.3389/fmicb.2022.1021325 (PMC9554438; doi:10.3389/fmicb.2022.1021325)
Supplement: Supplementary file 2 [file Data_Sheet_2.PDF]

Table S2. Metabolites uniquely detected in health, inflammation and CRC mice.

Feature ID	metabolite	m/z	RT	Formula	Library ID
4.99_861.4977 m/z	Hebevinoside VIII	861.4977	4.99	C47H74O15	HMDB0034598
3.47_146.0591 m/z	Indole-3-carboxaldehyde	146.0591	3.47	C9H7NO	C00294;C08493
3.41_164.0699 m/z	P-Acetaminobenzaldehyde	164.0699	3.41	C9H9NO2	NIST 231956
0.57_146.1648 m/z	Spermidine	146.1648	0.57	C7H19N3	HMDB0001257
0.93_331.0736 m/z	2-Amino-4-oxo-6-(1',2'-dioxoprolyl)-7,8-dihydroxypteridine	331.0736	0.93	C9H9N5O5	HMDB0001410
2.38_723.3679 m/z	Dalfopristin	723.3679	2.38	C34H50N4O9S	HMDB0015566
2.51_537.3884 m/z	(3b,5a,22E,24S)-Stigmasta-7,22,25-trien-3-ol 3-glucoside	537.3884	2.51	C35H56O6	HMDB0033089
1.91_183.1114 m/z	Benzyl glycinate	183.1114	1.91	C9H11NO2	HMDB0059934
2.62_195.1595 m/z	Ethylene oxide/propylene oxide copolymer	195.1595	2.62	CH4.[C2H4O]a.[C2H4O]a.[C3H6O]b	HMDB0032263
0.29_708.1097 m/z	Chymosin preparation, escherichia coli k-12	708.1097	0.29	C30H27N3O15	HMDB0032199
4.49_214.1538 m/z	Propenyl-L-NIO	214.1538	4.49	C10H19N3O2	
3.04_132.1011 m/z	6-Deoxyfagomine	132.1011	3.04	C6H13NO2	HMDB0036382
3.89_285.2027 m/z	Arginyl-Lysine	285.2027	3.89	C12H26N6O3	HMDB0028714
4.07_209.1278 m/z	Hydroxyprolyl-Isoleucine	209.1278	4.07	C11H20N2O4	HMDB0028866
3.41_103.1227 m/z	Cadaverine	103.1227	3.41	C5H14N2	HMDB0002322
4.64_285.1912 m/z	Melanostatin	285.1912	4.64	C13H24N4O3	HMDB0005764
1.16_192.0641 m/z	5-Hydroxyindoleacetic acid	192.0641	1.16	C10H9NO3	HMDB0000763
5.54_561.7636	CDP-	561.7636	5.54	C54H85N3O	HMDB0116036

m/z	DG(22:6(4Z,7Z,10Z,13Z,16Z,19Z)/20:2(11Z,14Z))			15P2	
1.72_465.3682 m/z	N-Stearoyl tyrosine	465.3682	1.72	C27H45NO4	HMDB0062343
1.10_549.4261 m/z	2-Hexaprenyl-6-methoxy-1,4-benzoquinol	549.4261	1.10	C37H56O3	HMDB0012148
1.89_338.0867 m/z	2,8-Dihydroxyquinoline-beta-D-glucuronide	338.0867	1.89	C15H15NO8	HMDB0011658
1.87_479.3838 m/z	Carpaine	479.3838	1.87	C28H50N2O4	HMDB0030272
0.95_535.4103 m/z	(3b,24R,25x)-26-Benzoyloxystigmast-5-ene-3-ol	535.4103	0.95	C36H54O3	HMDB0034553
1.71_926.7170 m/z	Beta-Elemonic acid	926.7170	1.71	C30H46O3	HMDB0034963
1.89_338.0867 m/z	2,8-Dihydroxyquinoline-beta-D-glucuronide	338.0867	1.89	C15H15NO8	HMDB0011658
4.99_861.4977 m/z	Hebevinoside VIII	861.4977	4.99	C47H74O15	HMDB0034598
3.15_314.1169 m/z	2-(4-Methyl-5-thiazolyl)ethyl octanoate	314.1169	3.15	C14H23NO2S	HMDB0032423*
3.40_773.2292 m/z	Kaempferol 3-[6'''-p-coumarylglucosyl-(1->2)-rhamnoside]	773.2292	3.40	C36H36O17	HMDB0040975*
1.71_750.4258 m/z	PS(14:1(9Z)/18:3(9Z,12Z,15Z))	750.4258	1.71	C38H66NO10P	HMDB0012348*

*Unique metabolites in CRC or inflammation mice
