# Supplementary material for: Integrative analysis of the mouse fecal microbiome and metabolome reveal dynamic phenotypes in the development of colorectal cancer
Source: Front Microbiol. 2022 Sep 28;13:1021325. doi: 10.3389/fmicb.2022.1021325 (PMC9554438; doi:10.3389/fmicb.2022.1021325)

Figure S1 Microbial α diversity in feces samples of group C and group BC. (A) Boxplots of Chao1 Richness Index. (B) Boxplots of Shannon Diversity Index. (C) Boxplots of Simpson Diversity Index. ns: $p > 0.05$, no significant difference.

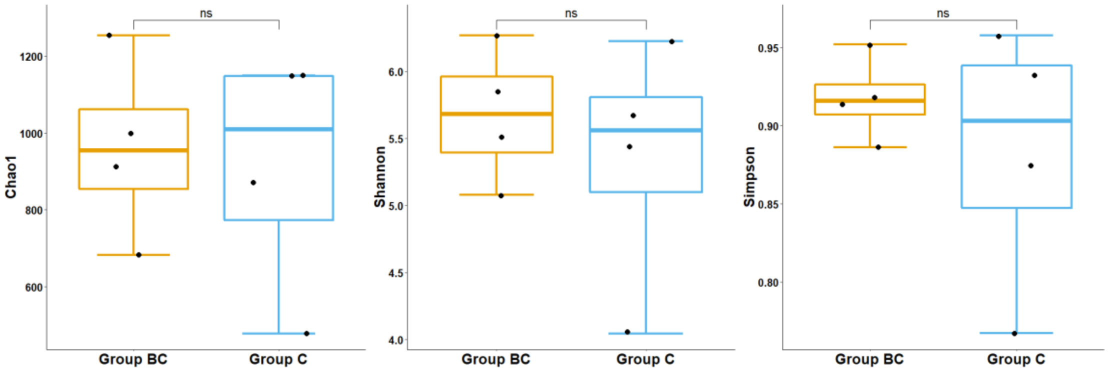

Supplement: Supplementary file 3 [file Data_Sheet_3.PDF]
