# Supplementary material for: Integrative analysis of the mouse fecal microbiome and metabolome reveal dynamic phenotypes in the development of colorectal cancer
Source: Front Microbiol. 2022 Sep 28;13:1021325. doi: 10.3389/fmicb.2022.1021325 (PMC9554438; doi:10.3389/fmicb.2022.1021325)

Figure S2 Microbial β diversity in feces samples of group C and group BC. PCoA plot of group C and group BC, there was no significant change in gut microbiota in group C and group BC.

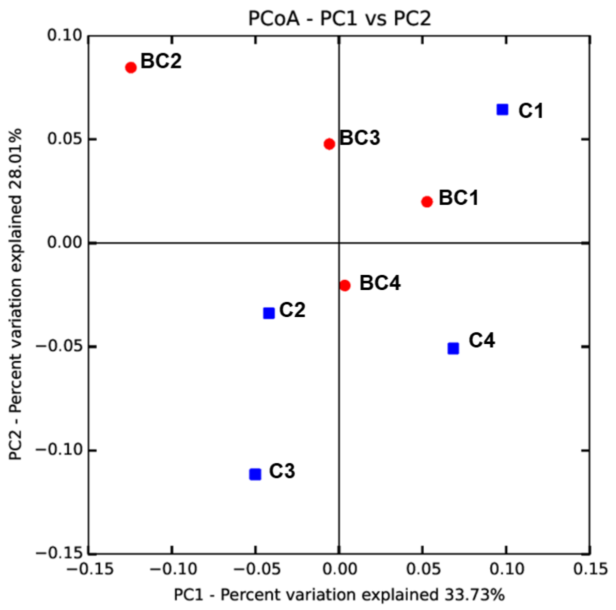

Supplement: Supplementary file 4 [file Data_Sheet_4.PDF]
