# Supplementary material for: Integrative analysis of the mouse fecal microbiome and metabolome reveal dynamic phenotypes in the development of colorectal cancer
Source: Front Microbiol. 2022 Sep 28;13:1021325. doi: 10.3389/fmicb.2022.1021325 (PMC9554438; doi:10.3389/fmicb.2022.1021325)

Figure S3 Hierarchical clustering analysis on the metabolite abundances in inflammation (A) and CRC (B).

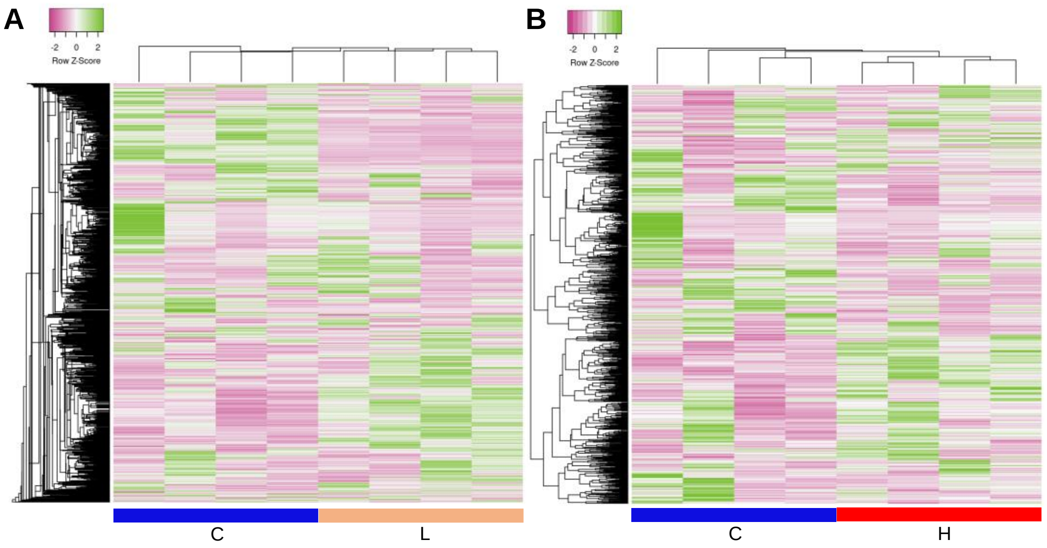

Supplement: Supplementary file 5 [file Data_Sheet_5.PDF]
