# Supplementary figures and images for: Integrative analysis of the mouse fecal microbiome and metabolome reveal dynamic phenotypes in the development of colorectal cancer
Source: Front Microbiol. 2022 Sep 28;13:1021325. doi: 10.3389/fmicb.2022.1021325 (PMC9554438; doi:10.3389/fmicb.2022.1021325)

Figure S4 Volcano plot of significantly changed fecal metabolites in CRC (A) and inflammation (B).

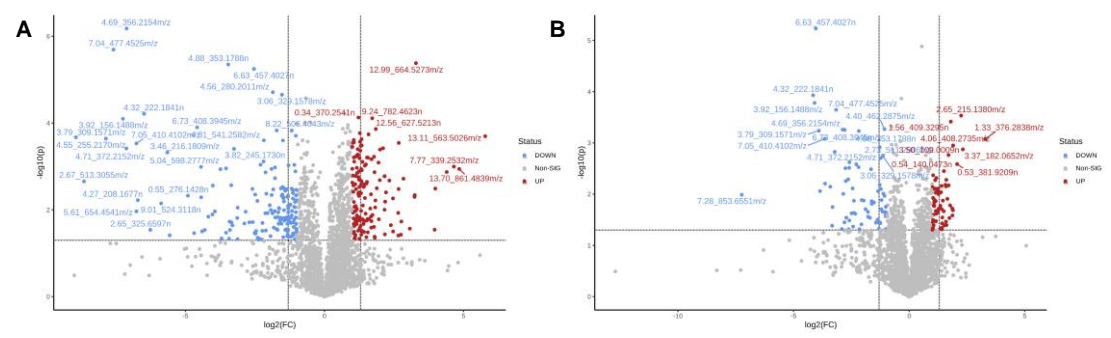

Supplement: Supplementary file 6 [file Data_Sheet_6.PDF]
